# Tackling brain drain at Chinese CDCs: understanding job preferences of public health doctoral students using a discrete choice experiment survey

**DOI:** 10.1186/s12960-022-00743-y

**Published:** 2022-05-23

**Authors:** Shimeng Liu, Yuanyuan Gu, Yi Yang, Elizabeth Schroeder, Yingyao Chen

**Affiliations:** 1 School of Public Health, Fudan University, 130 Dongan Rd, Xuhui, Shanghai, 200032 China; 2 NHC Key Laboratory of Health Technology Assessment (Fudan University), Shanghai, 200032 China; 3 Centre for the Health Economy, Macquarie University, Macquarie Park, NSW 2109 Australia; 4 Department of Health Systems and Populations, Macquarie University, Macquarie Park, NSW 2109 Australia

**Keywords:** Epidemiology and biostatistics, Doctoral students, Job preferences, Discrete choice experiment

## Abstract

**Background:**

Given the demands for public health and infectious disease management skills during COVID-19, a shortage of the public health workforce, particularly with skills and competencies in epidemiology and biostatistics, has emerged at the Centers for Disease Controls (CDCs) in China. This study aims to investigate the employment preferences of doctoral students majoring in epidemiology and biostatistics, to inform policy-makers and future employers to address recruitment and retention requirements at CDCs across China.

**Methods:**

A convenience sampling approach for recruitment, and an online discrete choice experiment (DCE) survey instrument to elicit future employee profiles, and self-report of their employment and aspirational preferences during October 20 and November 12, 2020. Attributes included monthly income, employment location, housing benefits, children’s education opportunities, working environment, career promotion speed and *bianzhi* (formally established post).

**Results:**

A total of 106 doctoral epidemiology and biostatistics students from 28 universities completed the online survey. Monthly income, employment location and *bianzhi* was of highest concern in the seven attributes measured, though all attributes were statistically significant and presented in the expected direction, demonstrating preference heterogeneity. Work environment was of least concern. For the subgroup analysis, employment located in a first-tier city was more likely to lead to a higher utility value for PhD students who were women, married, from an urban area and had a high annual family income. Unsurprisingly, when compared to single students, married students were willing to forgo more for good educational opportunities for their children. The simulation results suggest that, given our base case, increasing only monthly income from 10,000 ($ 1449.1) to 25,000 CNY ($ 3622.7) the probability of choosing the job in the third-tier city would increase from 18.1 to 53.8% (i.e., the location choice is changed).

**Conclusion:**

Monthly income and employment location were the preferred attributes across the cohort, with other attributes then clearly ranked and delineated. A wider use of DCEs could inform both recruitment and retention of a public health workforce, especially for CDCs in third-tier cities where resource constraints preclude all the strategies discussed here.

**Supplementary Information:**

The online version contains supplementary material available at 10.1186/s12960-022-00743-y.

## Background

The COVID-19 pandemic placed a spotlight on infectious disease prevention, identification, and population healthcare management [1]. Emerging lessons show that the quantity of available personnel and the quality of their expertise in overseeing the response and tracing systems are critical for managing a pandemic.

In China, the reform of the healthcare system and the development of a country-wide network of Centers for Disease Control (CDC) commenced in the early 2000s, in part due to reforms recommended from reviews of management of the SARS epidemic [2, 3]. One successfully accomplished reform was the formal establishment of Schools of Public Health (SPH) at major universities and medical schools, aiming to provide specialized training in different areas of public health (typically including epidemiology and biostatistics, environmental and occupational health, toxicology, nutrition and food hygiene, health inspection and quarantine, maternal and child health, and health policy and administration).

The “epidemiology and biostatistics” major proved the most popular, accounting for approximately 60% of SPH postgraduate student majors. The major was developed to provide skills and competencies for the CDC system in China [4]. Most employment positions in the CDCs are oriented towards graduates with this major, however, recent trends show that many epidemiology and biostatistics graduates have chosen to work elsewhere; for example in hospitals or pharmaceutical companies [5]. Recent research even suggested that employment at CDCs was their least preferred option [6]. Even worse, CDCs have been facing a high turnover in their workforce, in particular from those with stellar training in public health [7, 8], and for example, the number of personnel at CDCs had decreased by 4.5% from 2009 to 2018 [9]. The CDCs are therefore facing a significant brain drain crisis [5]. The design and implementation of policy interventions to improve the attractiveness of job prospects at the CDCs is now time critical, even more so in view of the current pandemic crisis.

This study used a discrete choice experiment (DCE) survey to elicit the job preferences of doctoral epidemiology and biostatistics students in China. A DCE uses a design where participants ‘trade’ options to reveal their preferences. We aimed to explore three questions: (1) what factors would they consider in their job choices? (2) what matters the most (ranking) in their aspirations? and (3) how much are they willing to pay for a desirable job feature? Effective policy interventions for job recruitment and retention need to simultaneously consider a variety of factors influencing the job decisions, which makes the DCE approach suitable for this research. The incorporation of a Willingness-to-Pay (WTP) component adds a magnitude of scale to our understanding, in addition to preference ranking. Finally, DCEs can be used to identify heterogeneity in preferences and could therefore be used to develop personalized incentive programs [10].

Evidence suggests that a number of DCE studies have already been conducted for student job preferences in China and many other countries, but not for public health students [11–13]. The current study is the first to investigate job preferences of public health doctoral students, in their capacity as the main source and resource for leadership in CDCs, and an older cohort most likely to have progressed in planning or establishing their careers. The purpose of this study is to robustly inform strategic and human resource planning in CDCs, and more broadly for the expansion of the public health workforce in China.

## Methods

### Sampling

Approximately 30 universities in China [6] currently offer epidemiology and biostatistics PhD degrees, and the total number being trained is approximately 300 students [6, 14, 15]. A voluntary anonymous web-based survey was created for data collection between October 20th and November 12th, 2020. A convenience sampling approach was adopted. We posted the survey link on *WeChat* (a popular Chinese social media site) and also sent the link to epidemiology and biostatistics PhD students at Chinese Universities identified by the authors. These students were asked to circulate the survey links to their classmates and to students in other universities they know. Two approaches were adopted to ensure the quality of survey data. First, we checked if the university a student stated they were affiliated with did deliver an epidemiology and biostatistics PhD program. Second, a DCE choice set was duplicated to see if the respondent would provide the same response (i.e., internal consistency).

The sample size was calculated using the approach proposed by Johnson and Orme [16], one of the most frequently used in the DCE literature [17]. It suggested that the minimal sample size for estimating the main effects model was 83. In order to conduct subgroup analyses, 100 respondents were targeted for data collection.

### Discrete choice experiment

Described by de Bekker-Grob et al. [10], a DCE approach is established contingent on a number of theories: (1) consumer theory; any job can be regarded as a given bundle of attributes [18]; (2) random utility theory; students likely opt for the employment alternative providing their highest utility [19]; and (3) experimental design theory, which generates choice sets in an efficient manner [20]. DCE is commonly used, is considered a realistic representation of actual decision-making and is shown to be one of the more robust methods to elicit preferences [21].

### Selection of attributes and their levels

Following DCE design guidelines [20], attributes were identified in a literature review [11–13, 22, 23] and in subsequent qualitative research (focus group discussion and in-depth interview). An initial set of ten attributes that incorporated personal and employment aspirations (with their levels) were identified, including monthly pre-tax income, *bianzhi*, employment location, housing benefits, children’s education opportunities, working environment, career promotion speed, workload, management style, and training opportunities. Here *bianzhi* is a unique job attribute in China—jobs with *bianzhi* are government-guaranteed positions with lifetime employment and the employees cannot be dismissed by their employers. In addition, *bianzhi* positions are considered to be an ‘iron rice bowl’ (‘job for life’) in China due to the guarantee of health insurance and pension [24, 25]. An iterative qualitative process was undertaken to finesse the attributes and levels. In-depth interviews were conducted with 6 doctoral students at Fudan University and Peking University. In review, ‘management style’ and ‘workload’ were removed at this stage. We also consulted three experts working in related public health trajectories, after which training opportunities were also removed and the pre-tax monthly income range was finalized to be set as 10,000–25,000 CNY, equivalent to $ 1449.1-$ 3622.7 (US$1 = CNY 6.901 in 2020 based on OECD data). This income range is consistent with the level of education and expertise of the survey respondents and their expected leadership roles within CDCs. The final set of attributes, their definitions, and their related levels are described in Table 1.

**Table 1:** Attributes and attribute levels

Attribute	Level	Description
Monthly income	10,000 CNY	Pre-tax salary
	15,000 CNY	
	20,000 CNY	
	25,000 CNY	
Employment location	First-tier city	The largest cities such as Beijing, Shanghai, Shenzhen, and Guangzhou
	Second-tier city	The medium-sized cities such as Qingdao and Xiamen
	Third-tier city	The minor cities such as Weihai and Yangzhou
Housing benefits	No housing benefits	Housing provided means a decent house is provided
	Housing allowance provided	
	Housing provided	
Children’ education opportunities	Ordinary	The educational opportunities available for children (including the elementary school, middle school or high school) in the workplace
	Good	
Career promotion speed	After 1 year	The number of years you would have to work before being eligible for promotion
	After 3 years	
	After 5 years	
Working environment	Ordinary	Management support, the relationship between supervisor and subordinate, amenities (such as regular bus, canteen and lounge), high-risk work environments and availability of equipment
	Better	
*bianzhi*	None	Jobs with *bianzhi* are government-guaranteed positions with lifetime employment and the employees cannot be dismissed by their employers. In addition, *bianzhi* positions are considered to be an ‘iron rice bowl’ in China due to the guarantee of health insurance and pension
	Offer	

According to the Organisation for Economic Co-operation and Development (OECD) data (https://data.oecd.org/conversion/exchange-rates.htm), the average annual exchange rate between US$ and CNY in 2020 was: US$1 = CNY 6.901

### Experimental design

Standard approaches were followed to achieve an unbiased, statistical efficiency in the design of the DCE [26]. Seven attributes were defined with three using 3 levels, three using 2 levels, and one using 4 levels, yielding a total of 864 (= 3 × 3 × 3 × 2 × 2 × 2 × 4) potential combinations. The design approach was informed by Huber and Zwerina [27], the DCE macros for SAS (version 9.4) were used for orthogonal main effect design, and selected profiles were organized into D-efficient choice designs (relative D-Efficiency: 77.9%). In total, 36 choice sets were identified and were further divided into three blocks to reduce cognitive burden. Within each version, a single choice set was duplicated to examine the internal consistency of respondent choices. All participants were randomized to receive one of the 3 versions according to their month of birth (Block 1: January to April; Block 2: May to August; Block 3: September to December). An example of the DCE choice set is provided in Additional file 1: Table S1.

### Survey

In addition to the DCE questions, the online questionnaire also collected information on student socio-demographic characteristics and career planning. The questionnaire was piloted between July and October 2020, to examine the comprehensibility, acceptability, and validity of the questionnaire with language and the layout revised thereafter.

The survey was prefaced with an explanation of the study purpose and construct, which also highlighted that their participation was voluntary, and that no identifiable data would be collected. The returned questionnaire indicates implied consent which is commonly adopted in anonymous online survey approaches. Ethics approval (Reference No. 2020-10-0853) was obtained from the ethics review board of the School of Public Health at Fudan University, and the research adhered to the tenets of the Declaration of Helsinki.

### Statistical analysis

STATA 15.1 was used for all analyses. Descriptive statistics were reported for participants’ socio-demographic characteristics. The utility (*U*) associated with a particular job is made up of two components: the deterministic component ${v}_{\mathrm{ni}}$ and the unobservable component ${\varepsilon }_{\mathrm{ni}}$. The utility function for the individual *n* associated with job *i* can be specified as:

$$\begin{gathered} U_{{{\text{ni}}}} = v_{{{\text{ni}}}} + \varepsilon_{{{\text{ni}}}} = \beta_{1} {\text{Location}}_{{{\text{Second}} - {\text{tier}}{\kern 1pt} {\text{city}}}} \hfill \\ + \beta_{2} {\text{Location}}_{{{\text{First}} - {\text{tier}}{\kern 1pt} {\text{city}}}} \hfill \\ + \beta_{3} {\text{Housing}}\,{\text{benefits}}_{{{\text{Allowance}}}} \hfill \\ + \beta_{4} {\text{Housing}}_{{{\text{Provided}}}} \hfill \\ + \beta_{5} {\text{Childrens}}\,{\text{education}}\,{\text{opportunities}}_{{{\text{Good}}}} \hfill \\ + \beta_{6} {\text{Career}}\,{\text{promotion}}\,{\text{speed}}_{{3{\text{year}}}} \hfill \\ + \beta_{7} {\text{Career}}\,{\text{promotion}}\,{\text{speed}}_{{1{\text{year}}}} \hfill \\ + \beta_{8} {\text{Working}}_{{3{\text{year}}}} \hfill \\ + \beta_{9} {\text{bianzhi}}_{{{\text{Yes}}}} + \beta_{10} {\text{Monthly}}\,{\text{income}} + \varepsilon_{{{\text{ni}}}} \hfill \\ \end{gathered}$$

Two econometric models were considered; the conditional logit (Clogit) and the mixed logit (MIXL) which uses random coefficients to accommodate potential unobserved preference heterogeneity [28]. The Akaike information criterion (AIC) and Bayesian information criterion (BIC) were used for model comparisons.

All attributes were coded to dummy variables. When estimating MIXL, all coefficients were specified as random (normally distributed) except monthly income which was fixed to facilitate a calculation of willingness to pay (WTP) [29–31], the relative monetary value that students place on various aspect of the job characteristics. WTP estimates are ratios of the coefficients between each attribute level and income attribute. The positive and negative results theoretically indicate to what extent participants would be willing to give up or to be compensated for a change in the attribute level. We also calculated the relative importance of each attribute as the proportion of the sum of its utility ranges [32]. Finally, we conducted a simulation study to understand to what extent the probability of choosing a given post would change as the levels of attributes changed.

## Results

### Study population

In total, 106 PhD students from 28 universities completed the online survey. We confirmed that each of these 28 universities has an epidemiology and biostatistics PhD program. Only 13 participants failed the internal consistency test, suggesting a very high level of engagement among the participants. The mean age was 28.0 years (SD = 3.2). The age ranged from 21 to 40, and 93.4% of them were older than 24 which is sensible given the stage of their study. Most were female (67.0%), single (78.3%), and from urban areas (57.0%). In China, the majority of public health students are female [33]. Around 88.7% of the students wanted to take epidemiology or biostatistics-related jobs after graduation. See Table 2 for more details.

**Table 2:** Respondent characteristics

*n* = 106	*n*	%
Age(year), Mean ± SD	28.0(± 3.2)	
Gender		
Male	35	33.0
Female	71	67.0
Place of origin		
Rural	46	43
Urban	60	57
Marital status		
Single	83	78.3
Married	23	21.7
Monthly consumption (CNY)^a^		
< 1500	20	18.9
1500–2500	45	42.5
2500–3500	18	17.0
3500–4500	7	6.6
4500–5500	2	1.9
> 5500	14	13.2
Annual family income (CNY)		
< 50,000	15	14.2
50,000–100,000	34	32.1
100,000–150,000	16	15.1
150,000–200,000	13	12.3
200,000–250,000	11	10.4
250,000–300,000	4	3.8
> 300,000	13	12.3
Will you take a job related to your major after graduation?		
Yes	94	88.7
No	1	0.9
Not sure	11	10.4
Career planning (multiple-choice: frequency)		
University or scientific research institution	76	29.1
Hospital	62	23.8
CDC	35	13.4
Government agency	41	15.7
Pharmaceutical company	43	16.5
Others	4	1.5

*SD* standard deviation, *CNY* Chinese yuan, *CDC* Centers for Disease Control and Prevention. ^a^According to the Organization for Economic Co-operation and Development (OECD) data (https://data.oecd.org/conversion/exchange-rates.htm), the average annual exchange rate between US$ and CNY in 2020 was: US$1 = CNY 6.901

### DCE results

The DCE results reported were all based on the full sample (1272 observations from 106 students). A sensitivity analysis was undertaken excluding the 13 participants who failed the internal consistency test (Additional file 1: Table S2), though these changes did not materially affect the findings. It was also suggested that irrational responses from DCEs should not be discarded [34]. The AIC and BIC values suggested the MIXL was preferable to Clogit. As such, the main paper reports the MIXL estimates (Table 3) and the Clogit estimates are presented in Additional file 1: Table S3.

**Table 3:** Mixed logit estimates and WTP (*n* = 106)

Attributes and levels	Coefficient (SE)	*p-*value	SD (SE)	*p-*value	WTP (CNY)^a^	95% CI	
Employment location (ref: third-tier city)							
Second-tier city	0.814 (0.157)	*p < *0.001	0.805 (0.207)	*p < *0.001	7357.3	4726.2	10,360.6
First-tier city	1.508 (0.215)	*p < *0.001	1.444 (0.229)	*p < *0.001	13,638.2	10,088.1	18,184.4
Housing benefits (ref: no housing benefits)							
Housing allowance provided	0.419 (0.135)	*p = *0.002	0.198 (0.326)	*p = *0.544	3792.5	1439.2	6231.9
Housing provided	0.715 (0.139)	*p < *0.001	0.285 (0.299)	*p = *0.341	6466.9	4103.1	9293.4
Children’s education opportunities (ref: ordinary)							
Good	0.584 (0.108)	*p < *0.001	0.498 (0.163)	*p = *0.002	5278.1	3367.8	7502.6
Career promotion speed (ref: 5 years)							
3 years	0.243 (0.129)	*p = *0.060	0.180 (0.444)	*p = *0.685	2200.7	-85.3	4656.5
1 year	0.584 (0.141)	*p < *0.001	0.522 (0.232)	*p = *0.024	5280.0	2852.6	7983.5
Working environment (ref: ordinary)							
Better	0.193 (0.091)	*p = *0.033	0.221 (0.238)	*p = *0.354	1748.0	123.6	3403.9
*bianzhi* (ref: none)							
Offer	0.824 (0.134)	*p < *0.001	0.831 (0.157)	*p < *0.001	7447.6	5153.6	10,091.5
Monthly income	0.0001106 (0.0000122)	*p < *0.001					
LR Chi^2^ (10)	69.48						
Number of observations	1272						
Log-likelihood	− 662.25						
AIC	1362.50						
BIC	1473.49						

*WTP* willingness to pay, *CNY* Chinese yuan, *SD* standard deviation, *SE* standard error, *95% CI* 95% confidence intervals, *AIC* Akaike information criterion, *BIC* Bayesian information criterion ^a^US$1 = CNY 6.901

**Fig. 1:**
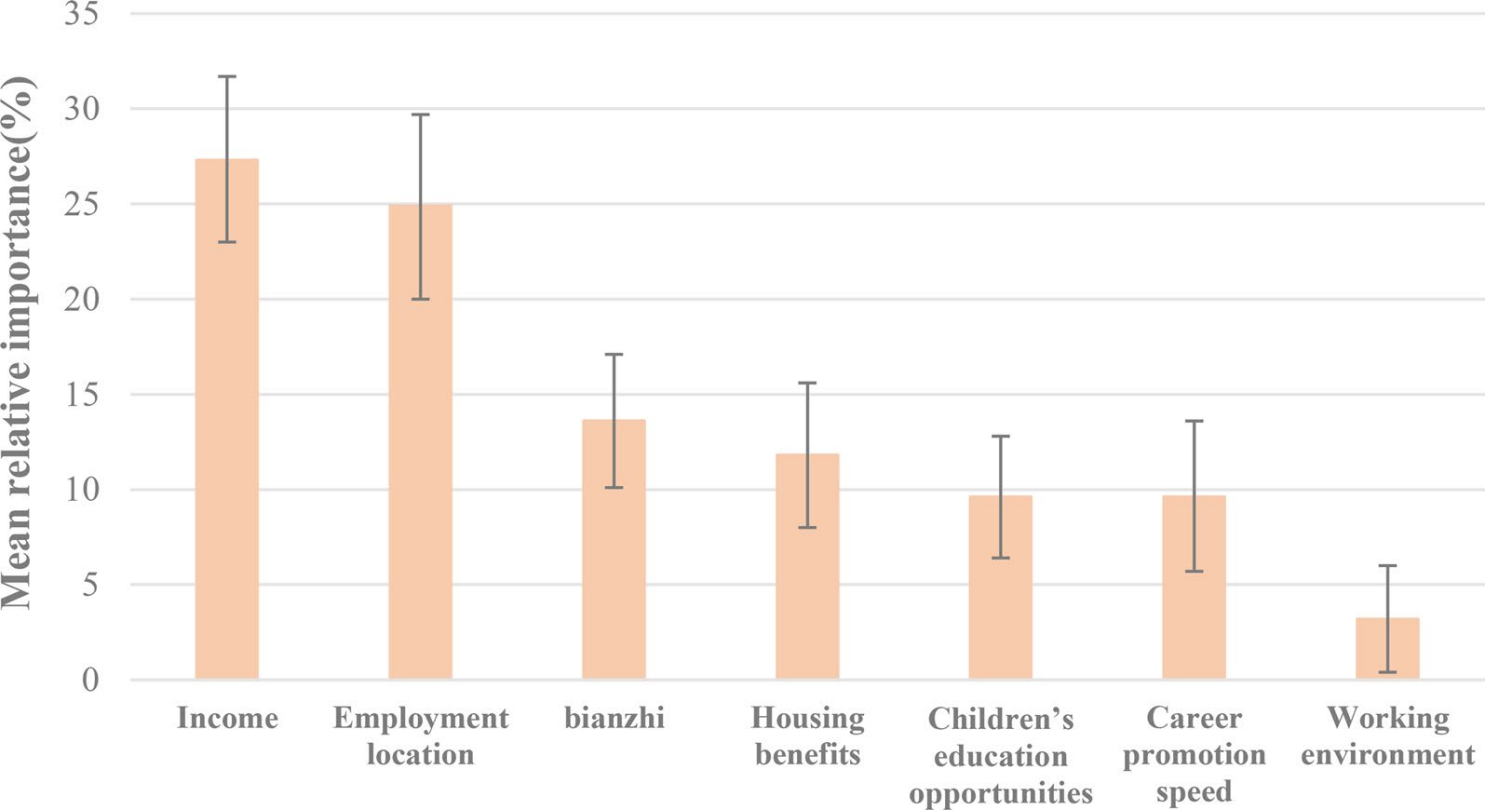
Mean relative importance of the attributes

Statistical significance of all the mean preference parameters suggests that the selected attributes are all significant predictors of the job choice. Some estimated standard deviations are significant, indicating the existence of preference heterogeneity. The students particularly cared about the employment location, strongly favoring first-tier city over third-tier city (β = 1.508; *p* < 0.001). They also exhibited strong preferences for a job with *bianzhi* compared to one without (β = 0.824; *p* < 0.001), and for a job with housing provide compared to one without any housing benefits (β = 0.715; *p* < 0.001). Their preference for career promotion speed (after 1 year compared to after 5 years) and children education opportunities (good compared to ordinary) turned out to be the same (β = 0.584; *p* < 0.001). Good working environment also mattered, but to a lesser extent (β = 0.401; *p = *0.033). The relative importance shown in Fig. 1 indicates that students gave most importance to income (score of 27.3%) and employment location (score of 24.9%) which clearly dominated the other attributes including *bianzhi*, housing benefits, children’s education opportunities, career promotion speed. Working environment was deemed the least important with a score of 3.2%.

The WTP analysis quantified how much salary the students were willing to sacrifice for a desired level of a job attribute compare with the reference level. WTP estimates are documented in Table 3. Students were willing to forgo 13,638.2 CNY ($ 1976.3) in the monthly salary for a job located in the first-tier city over a third-tier city, 7447.6 CNY ($ 1079.2) for a job with *bianzhi* over one without, 6466.9 CNY ($ 937.1) to get a job with housing provided over one without any housing benefit, 5280.0 CNY ($765.1) for a job with a promotion opportunity after 1 year over one with promotion opportunity after 5 years, 5278.1 CNY ($ 764.8) for a job with good children education opportunities over one without, and finally 1748.0 CNY ($ 253.3) for a job with good working environment over one without.

**Fig. 2:**
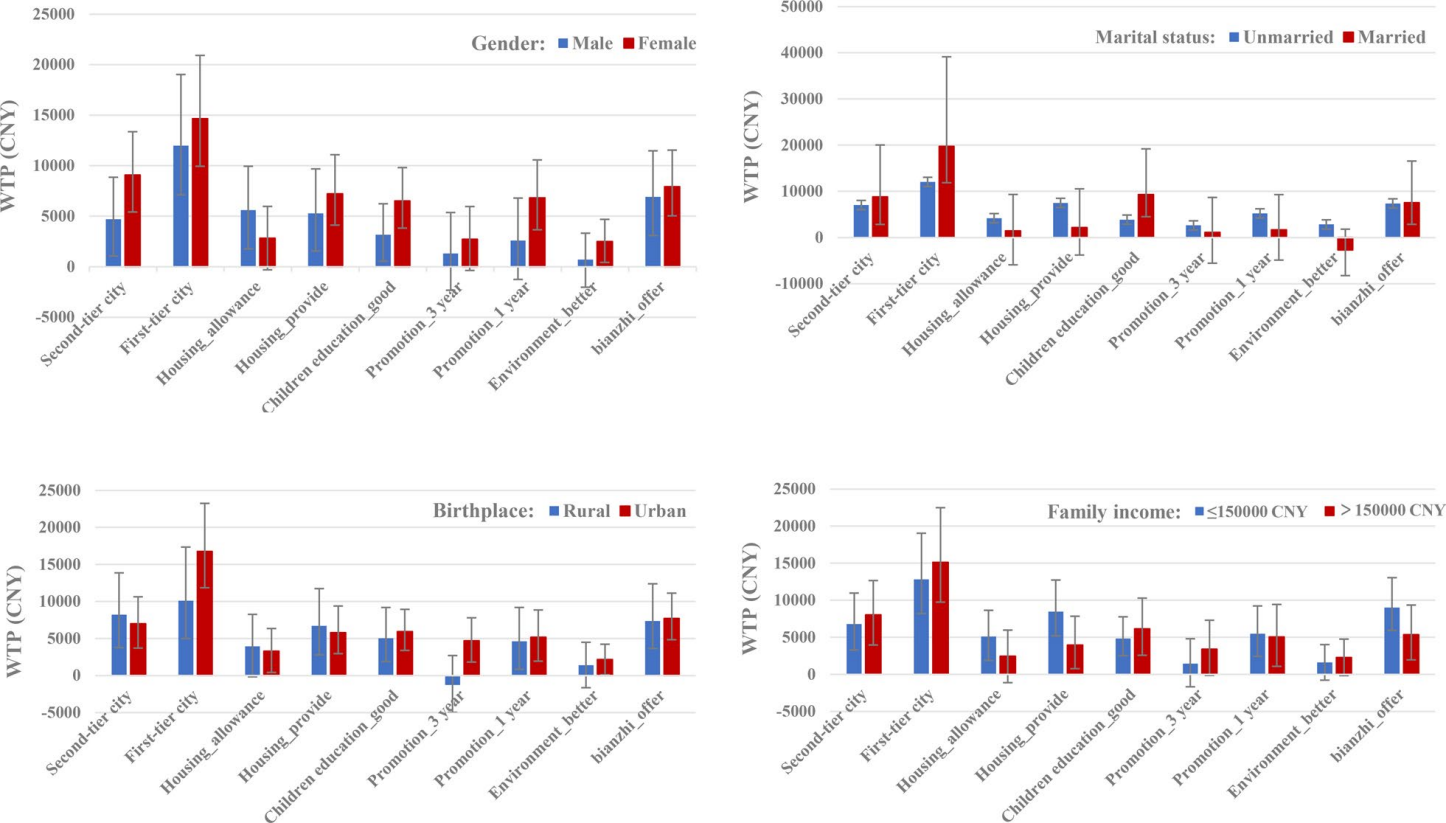
Willingness-to-pay estimation for subgroup population

The results of subgroup analyses are presented in Additional file 1: Table S4 and Fig. 2. For the subgroups, most of the attributes remained statistically significant in influencing students job preferences. Based on the WTP estimates, it can be seen that the job in first-tier city was more likely to lead to a higher utility value for respondents who were women, married, with high annual family income and from urban area. Predictably, compared to the single students, married were willing to forgo more income for better opportunities for their children’s education.

### Scenario analysis

**Fig. 3:**
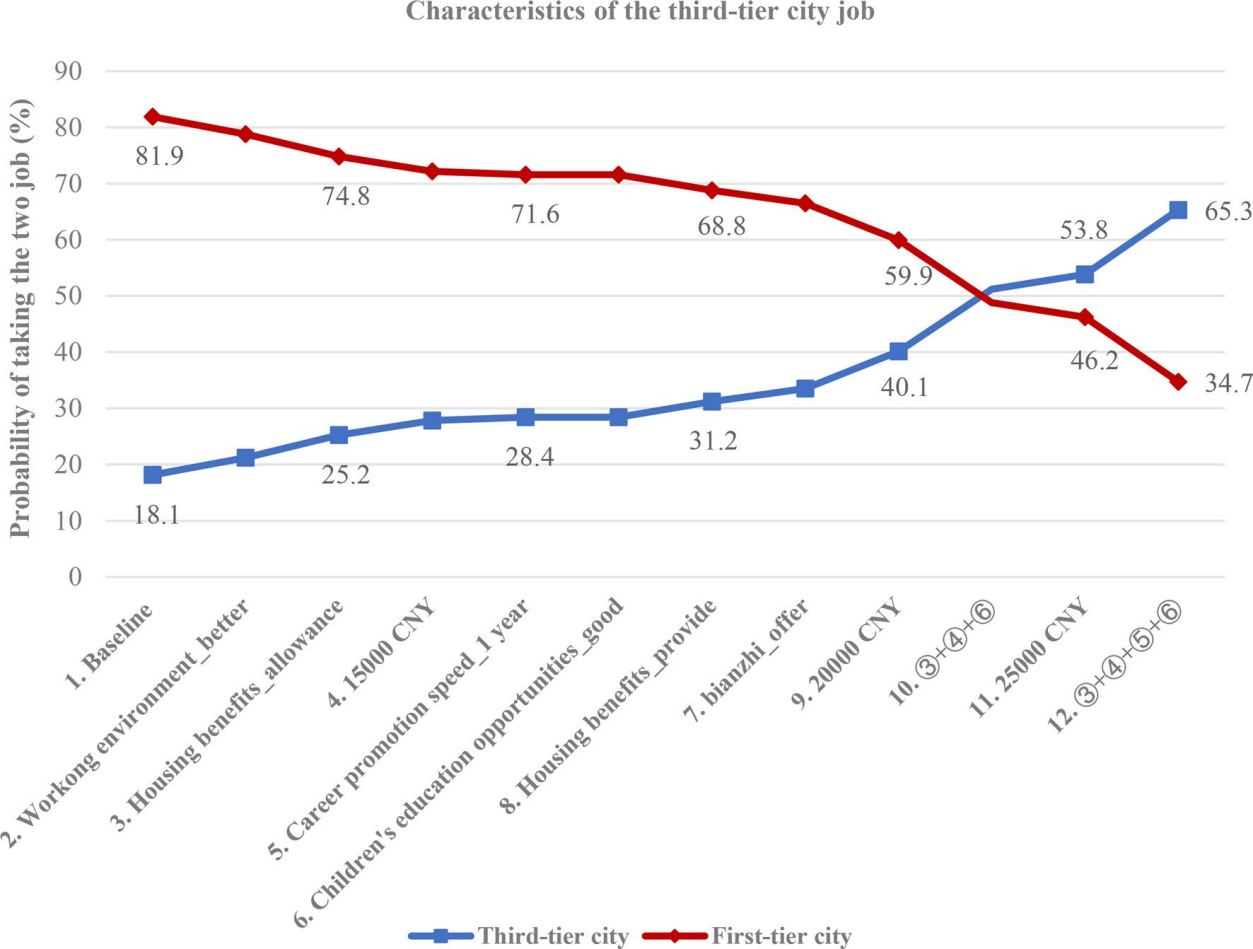
Simulated preferences for job posting under various potential policy scenarios. Changes in the probabilities of taking a job, third-tier city versus first-tier city, as conditions in the third-tier city job improve

The simulation results are shown in Fig. 3. The baseline setting is that two job descriptions only differ in employment location and with all attributes set to reference levels (10,000 CNY ($ 1449.1) monthly income; no housing benefits; ordinary children’s education opportunities; promotion after 5 year; no *bianzhi*, ordinary working environment). In this case, the probability of taking a job in the third-tier city is 18.1% compared to a job in the first-tier city of 81.9%—the latter substantially dominates the former. When increasing only monthly income from 10,000 ($ 1449.1) to 25,000 CNY ($ 3622.7) the probability of choosing the job in the third-tier city increases to 53.8%, and reflects sensitivity in magnitude. The incentive combination “③ + ④ + ⑤ + ⑥” further increases the preference for a third-tier city job to 65.3%.

## Discussion

In grappling to manage the COVID-19 pandemic, the quality of the public health workforce has magnified in importance, as is the perceived value of their role in CDCs. Attracting and retaining the talent, skills and competencies and reducing staff turnover in CDCs is therefore critical to address. This article intended to inform aspects of this complex policy issue by undertaking a DCE survey with likely graduates, the PhD students with the epidemiology and biostatistics major.

In the short period after the COVID-19 outbreak, the respect for the CDC workforce was significantly enhanced. This occurred against a backdrop of a trend of a decreasing workforce experiencing flat wages, and a noticeably widening income gap between the disease control workforce and staff in other roles and workplaces [35], exacerbating recruitment and retention. Our study confirms that financial incentives are still the most important lever for recruitment and retention, and when compared across employment locations, the magnitude of the incentive is effectual. As such, a 5000 CNY ($ 724.5) salary increase from baseline was relatively ineffective, but became significant when further increased (Fig. 3). The relationship between monetary incentive and choice of rural or remote practice by health professionals is likely to be highly context specific. For example, the study conducted in Norway (a high-income country) by Holte demonstrated that an increase in income from 10 to 20% had no impact on young doctors’ job choice [36], while one study conducted in Kenya (a low-income country) indicated that an increase in salary from 10 to 20% could incentivize health workers to choose a rural posting [37].

Among non-monetary attributes, working in the first-tier cities had the largest impact. Large metropolitan centers offer more career and educational advancement, better employment prospects, and easier access to lifestyle-related services and amenities. Studies from other countries have reported that the more urban the job, the more it will be preferred [38, 39]. In addition, students from urban areas showed a much stronger preference to work in the city. Therefore, as one possible emergent option, attracting and retaining students from a rural background for the grassroots CDCs might be more effective.

Contrary to the previous research with heath administration [22] or nursing students [40] which found that *bianzhi* had the lowest utility in job preferences, *bianzhi* turned out to be the second most important non-monetary factor in this study. In China, a job with *bianzhi* will likely accrue better job security [24, 25]. Respondents in this study were older and some of them had families, so *bianzhi* may matter to them much more. This suggests that, to avoid brain drain for the CDC system, it is necessary to prepare positions with *bianzhi* for the more important roles. Given the fixed amount of *bianzhi*, this means a dynamic adjustment and reallocation mechanism could be implemented in the CDC system. The allocation of public health workforce in CDCs should achieve the standard of 1.75 per 10,000 population recommended by the Chinese National Health Commission (only 1.42 per 10,000 population in 2017), and the quantity of *bianzhi* allocated for high-level public health talents may increase based on needs.

The provision of affordable housing was highly valued, consistent with other studies [41]. In recent years, the Chinese housing market has boomed in cities and housing ownership has become unaffordable for many families. The Chinese government has adhered to the policy that ‘houses are used for living, not for speculation’, and local governments have also implemented numerous interventions, such as restricting the purchase of rental houses, increasing the supply of affordable housing, but the housing price has still become unaffordable to citizens [42]. Constrained by their financial capacity, the CDCs at third-tier cities may not be able to provide housing for their employees but housing benefits, coupled with other incentives such as good educational resources may work equally well. Other research also found that a bundle of incentives, such as housing combined with educational opportunities or improved working environment, are more likely to be effective in retaining health workers in the long term [43].

Career promotion speed is another important non-monetary factor. Rapid environmental changes and increasingly fiercer competition have forced students to focus not only immediate decisions, but also their longer-term career opportunities. Similar results have been reported in other human resource DCE studies in low- and middle-income countries [41, 44] and another study conducted in China found that the most important factor influencing job satisfaction in CDCs was personal development [45]. This suggests that developing clear career paths for CDC posts and adopting strategies to increase public recognition or policy influence should be considered.

The children education opportunities attribute had a relatively smaller effect on job preference, contrary to a study conducted in Nepal [46] in which children’s education was found to be much stronger predictors of choice. Given our sample’s demographics and cultural preferences, it may be that most of the students in our study had not started a family, so children’s education was not a pre-eminent concern. Our subgroup analysis strengthened this assumption as the married PhD students had a stronger preference for the children’s education opportunities.

Working environment was the least important factor. In our study, working environment refers to management support, the relationship between supervisor and subordinate, amenities (such as regular bus, canteen and lounge), high-risk work environments and availability of equipment. This finding is consistent with the results of an earlier quantitative study in which working environment was not considered as a major contributing factor towards job choice in China [47]. In addition, an early study from Scotland also found that health workers place a lower weight on having good relations with staff [48]. It suggests that changing the working environment may not be an effective intervention to improve recruitment and retention problems at CDCs.

### Limitations

The study has several limitations. First, the generalizability of the study findings may be limited by the convenience sampling approach. It is not possible to identify the statistics of the target population of epidemiology and biostatistics PhD students currently being trained at universities in China so the representativeness of our sample could not be fully assessed. However, our sample size accounted for around one-third of the total, and 28 out of 30 universities. The key sample statistics were also sensible, most being female and older than 24. It should also be noted that, while 106 students seemed to be a relatively small sample, each had responded to 12 choice questions resulting in a total sample size of 1272 choice observations for data analysis. The mean preference coefficients (as shown in Table 3) were mostly highly significant. Second, we could not calculate the response rate of our survey. The response rate is an important indicator of the quality of a survey study. With a low response rate, the results can be biased. We posted the survey link on WeChat and also sent the link to epidemiology and biostatistics PhD students at Chinese Universities identified by our research team. These students were asked to circulate the survey links to their classmates and to students in other universities they know. While this approach allowed us to reach as many students as possible, it also means that we would not be able to find out how many students had clicked on our survey link and thus seen our invitation. Nevertheless, our response rate is at least around 35% assuming all the targeted students saw out invitation and we consider the actual response rate for students is likely much higher. Also, our study is less likely to suffer from selection bias given there are no obvious reasons for anyone to reject our invitation unless they were very busy at the time of survey which itself is of random nature. Third, it was not possible to include a larger number of attributes because the trade of options would become overwhelming to respondents who are less willing to critically appraise attributes as the burden of data completion grows. Not all potentially important attributes, such as workload, were assessed in our study though we attempted to ensure we addressed the key factors, and attributes such as workload were rolled up into working environment. Fourth, respondents in this study were not limited to the final year epidemiology and biostatistics PhD students. Though job preferences may vary across PhD students at different levels, given the limited number of students available, we were unable to examine these differences.

## Conclusions

To the best of our knowledge, this is the first study to investigate job preferences of epidemiology and biostatistics PhD students in China. Monthly income and work location were the most important attributes that impact the student’s job choices. Results from this study will provide policy-makers with information to develop more effective policies to improve job attractiveness and retention at CDCs, especially those located in third-tier cities. Further qualitative research such as in-depth interviews and focus group discussions with PhD students will be required to determine the specific reasons for job choices, and broader literature searches can inform a breadth of innovative options for workforce planning, accommodating for the complexity of incentives required to address workforce shortages.

## Supplementary Information

**Additional file 1: Table S1.** An example choice set. **Table S2.** Mixed logit estimates (Excluded the participants who failed the internal consistency test, n = 93). **Table S3.** Conditional logit estimates (n = 106). **Table S4.** Subgroup analyses.

## Data Availability

The data used and/or analyzed during the study are available from the corresponding author on reasonable request.

## Abbreviations

COVID-19: Coronavirus disease 2019
CDC: Center for Disease Control and Prevention
DCE: Discrete choice experiment
SD: Standard deviation
SE: Standard error
WTP: Willingness to pay
