# Supplementary material for: Tackling brain drain at Chinese CDCs: understanding job preferences of public health doctoral students using a discrete choice experiment survey
Source: Hum Resour Health. 2022 May 23;20:46. doi: 10.1186/s12960-022-00743-y (PMC9125964; doi:10.1186/s12960-022-00743-y)
Supplement: Supplementary file 1 — Additional file 1: Table S1. An example choice set. Table S2. Mixed logit estimates (Excluded the participants who failed the internal consistency test, n = 93). Table S3. Conditional logit estimates (n = 106). Table S4. Subgroup analyses. [file 12960_2022_743_MOESM1_ESM.docx]

**Table S1** An example choice set

**Table S2** Mixed logit estimates (Excluded the participants who failed the internal consistency test, n=93)

**Table S3** Conditional logit estimates (n=106)

**Table S4** Subgroup Analyses

**Table S1** An example choice set

| **Attributes/levels** | **Job 1** | **Job 2** |
| --- | --- | --- |
| Monthly income | 25000 CNY | 10000 CNY |
| Employment location | Second-tier city | First-tier city |
| Housing benefits | Housing provided | No housing benefits |
| Children’ education opportunities | Ordinary | Good |
| Career promotion speed | After 3 years | After 1 year |
| Working environment | Ordinary | Better |
| *bianzhi* | Offer | None |
| **Which job would you prefer?** |  |  |

**Table S2** Mixed logit estimates (Excluded the participants who failed the internal consistency test, n=93)

| **Attributes and levels** | **β** | **SE** | **SD** | **SE** |
| --- | --- | --- | --- | --- |
| **Employment location** (ref: Third-tier city) |  |  |  |  |
| Second-tier city | 0.830*** | 0.167 | 0.595**** | 0.285 |
| First-tier city | 1.677*** | 0.245 | 1.487***** | 0.279 |
| **Housing benefits** (ref: No) |  |  |  |  |
| Housing allowance provided | 0.408*** | 0.150 | 0.076 | 0.351 |
| Housing provided | 0.733*** | 0.156 | 0.204 | 0.408 |
| **Children’s education opportunities** (ref: Ordinary) |  |  |  |  |
| Good | 0.676*** | 0.122 | 0.360 | 0.228 |
| **Career promotion speed** (ref: 5 year) |  |  |  |  |
| 3 year | 0.244*** | 0.141 | 0.165 | 0.611 |
| 1 year | 0.528***** | 0.160 | 0.736***** | 0.206 |
| **Working environment** (ref: Ordinary) |  |  |  |  |
| Better | 0.212**** | 0.104 | 0.330 | 0.226 |
| ***bianzhi*** (ref: No) |  |  |  |  |
| Offer | 0.918***** | 0.101 | 0.905***** | 0.166 |
| **Income** | 0.0001216***** | 0000144 |  |  |
| LR chi2(9) | 69.28 | | | |
| Number of obs | 1116 | | | |
| Log likelihood | -552.280 | | | |

**P* < 0.10; ***P* < 0.05; ****P* < 0.01

**Table S3** Conditional logit estimates (n=106)

| **Attributes and levels** | **β** | **SE** |
| --- | --- | --- |
| **Employment location** (ref: Third-tier city) |  |  |
| Second-tier city | 0.602***** | 0.114 |
| First-tier city | 1.033***** | 0.144 |
| **Housing benefits** (ref: No) |  |  |
| Housing allowance provided | 0.299***** | 0.100 |
| Housing provided | 0.498***** | 0.105 |
| **Children’s education opportunities** (ref: Ordinary) |  |  |
| Good | 0.413***** | 0.073 |
| **Career promotion speed** (ref: 5 year) |  |  |
| 3 year | 0.161*** | 0.092 |
| 1 year | 0.388***** | 0.104 |
| **Working environment** (ref: Ordinary) |  |  |
| Better | 0.126*** | 0.066 |
| ***bianzhi*** (ref: No) |  |  |
| Offer | 0.554***** | 0.079 |
| **Income** | 0.000081(7.37e-06) ***** |  |
| Wald chi2(10) | 190.31 |  |
| Number of observations | 1272 |  |
| Log likelihood | -696.989 |  |
| AIC | 1413.978 |  |
| BIC | 1472.393 |  |

**P* < 0.10; ***P* < 0.05; ****P* < 0.01

**Table S4** Subgroup Analyses

| **Attributes and levels** | **Male (n=35)** | | | | | | | | | | | | | **Female (n=71)** | | | | | | | | | | | | | | | |
| --- | --- | --- | --- | --- | --- | --- | --- | --- | --- | --- | --- | --- | --- | --- | --- | --- | --- | --- | --- | --- | --- | --- | --- | --- | --- | --- | --- | --- | --- |
|  | **Coefficient (SE)** | | ***p* value** | | | **SD (SE)** | | | | ***p* value** | | | | **Coefficient (SE)** | | | | ***p* value** | | | | **SD (SE)** | | | | ***p* value** | | | |
| Second-tier city | 0.603(0.246) | | .01 | | | 0.431(0.477) | | | | .367 | | | | 0.960(0.212) | | | | <.001 | | | | 0.974(0.285) | | | | .001 | | | |
| First-tier city | 1.537(0.349) | | <.001 | | | 1.289(0.411) | | | | .002 | | | | 1.548(0.279) | | | | <.001 | | | | 1.510(0.305) | | | | <.001 | | | |
| Housing allowance provided | 0.718(0.260) | | .006 | | | 0.557(0.376) | | | | .138 | | | | 0.297(0.165) | | | | .07 | | | | 0.100(0.327) | | | | .76 | | | |
| Housing provided | 0.676(0.251) | | .007 | | | 0.201(0.622) | | | | .747 | | | | 0.763(0.174) | | | | <.001 | | | | 0.240(0.359) | | | | .50 | | | |
| Children’s education opportunities: Good | 0.408(0.168) | | .02 | | | 0.007(0.426) | | | | .987 | | | | 0.688(0.146) | | | | <.001 | | | | 0.684(0.189) | | | | <.001 | | | |
| Career promotion speed: 3 year | 0.167(0.233) | | .47 | | | 0.340(0.379) | | | | .369 | | | | 0.288(0.163) | | | | .08 | | | | 0.237(0.364) | | | | .52 | | | |
| Career promotion speed: 1 year | 0.331(0.243) | | .17 | | | 0.675(0.349) | | | | .053 | | | | 0.720(0.174) | | | | <.001 | | | | 0.421(0.331) | | | | .20 | | | |
| Working environment: Better | 0.092(0.165) | | .58 | | | 0.309(0.364) | | | | .396 | | | | 0.265(0.111) | | | | .02 | | | | 0.011(0.827) | | | | .98 | | | |
| *bianzhi:* Offer | 0.888(0.266) | | <.001 | | | 1.080(0.311) | | | | .001 | | | | 0.837(0.162) | | | | <.001 | | | | 0.710(0.183) | | | | <.001 | | | |
| Income | 0.0001281(0.000023) | | <.001 | | |  | | | |  | | | | 0.0001056(0.000015) | | | | <.001 | | | |  | | | |  | | | |
|  | **Unmarried (n=83)** | | | | | | | | | | | | | | | | **Married (n=23)** | | | | | | | | | | | | |
|  | **Coefficient (SE)** | | | ***p* value** | | | | | **SD (SE)** | | | | ***p* value** | | | | **Coefficient (SE)** | | | | ***p* value** | | | | **SD (SE)** | | | | ***p* value** |
| Second-tier city | 0.840(0.178) | | | <.001 | | | | | 0.720(0.231) | | | | .002 | | | | 0.887(0.409) | | | | .03 | | | | 1.099(0.488) | | | | .02 |
| First-tier city | 1.439(0.239) | | | <.001 | | | | | 1.394(0.252) | | | | <.001 | | | | 2.270(0.567) | | | | <.001 | | | | 1.481(0.548) | | | | .007 |
| Housing allowance provided | 0.502(0.150) | | | .001 | | | | | 0.120(0.468) | | | | .80 | | | | 0.419(0.354) | | | | .24 | | | | 0.016(0.474) | | | | .97 |
| Housing provided | 0.895(0.164) | | | <.001 | | | | | 0.075(0.490) | | | | .88 | | | | 0.520(0.349) | | | | .14 | | | | 0.364(0.549) | | | | .51 |
| Children’s education opportunities: Good | 0.463(0.107) | | | <.001 | | | | | 0.329(0.198) | | | | .10 | | | | 0.880(0.354) | | | | .01 | | | | 1.121(0.455) | | | | .01 |
| Career promotion speed: 3 year | 0.315(0.151) | | | .04 | | | | | 0.389(0.285) | | | | .17 | | | | 0.009(0.331) | | | | .98 | | | | 0.030(0.397) | | | | .94 |
| Career promotion speed: 1 year | 0.625(0.143) | | | <.001 | | | | | 0.215(0.339) | | | | .53 | | | | 0.435(0.433) | | | | .32 | | | | 1.145(0.569) | | | | .04 |
| Working environment: Better | 0.341(0.106) | | | .001 | | | | | 0.260(0.237) | | | | .27 | | | | 0.258(0.225) | | | | .25 | | | | 0.080(0.453) | | | | .86 |
| *bianzhi:* Offer | 0.882(0.152) | | | <.001 | | | | | 0.723(0.157) | | | | <.001 | | | | 0.896(0.369) | | | | .02 | | | | 1.118(0.004) | | | | 0.393 |
| Income | 0.0001192(0.000014) | | | <.001 | | | | |  | | | |  | | | | 0.0001071(0.000029) | | | | <.001 | | | |  | | | |  |
|  | **Rural (n=46)** | | | | | | | | | | | | | | **Urban (n=60)** | | | | | | | | | | | | | | |
|  | **Coefficient (SE)** | ***p* value** | | | | | **SD (SE)** | | | | ***p* value** | | | | **Coefficient (SE)** | | | | ***p* value** | | | | **SD (SE)** | | | | ***p* value** | | |
| Second-tier city | 0.790(0.227) | .001 | | | | | 0.808(0.302) | | | | .007 | | | | 0.977(0.259) | | | | <.001 | | | | 0.928(0.366) | | | | .011 | | |
| First-tier city | 0.970(0.261) | <.001 | | | | | 1.161(0.286) | | | | <.001 | | | | 2.339(0.420) | | | | <.001 | | | | 1.873(0.413) | | | | <.001 | | |
| Housing allowance provided | 0.379(0.199) | .06 | | | | | 0.377(0.337) | | | | .26 | | | | 0.458(0.204) | | | | .02 | | | | 0.022(0.519) | | | | .97 | | |
| Housing provided | 0.644(0.202) | .001 | | | | | 0.198(0.407) | | | | .63 | | | | 0.808(0.211) | | | | <.001 | | | | 0.390(0.405) | | | | .34 | | |
| Children’s education opportunities: Good | 0.483(0.151) | .001 | | | | | 0.505(0.237) | | | | .03 | | | | 0.828(0.192) | | | | <.001 | | | | 0.629(0.240) | | | | .009 | | |
| Career promotion speed: 3 year | -0.121(0.187) | .52 | | | | | 0.030(0.715) | | | | .97 | | | | 0.683(0.214) | | | | .001 | | | | 0.048(0.474) | | | | .92 | | |
| Career promotion speed: 1 year | 0.446(0.188) | .02 | | | | | 0.292(0.441) | | | | .51 | | | | 0.722(0.233) | | | | .002 | | | | 0.938(0.298) | | | | .002 | | |
| Working environment: Better | 0.135(0.142) | .34 | | | | | 0.425(0.237) | | | | .07 | | | | 0.301(0.144) | | | | .04 | | | | 0.241(0.274) | | | | .38 | | |
| *bianzhi:* Offer | 0.707(0.185) | <.001 | | | | | 0.785(0.217) | | | | <.001 | | | | 1.076(0.230) | | | | <.001 | | | | 0.986(0.246) | | | | <.001 | | |
| Income | 0.000096(0.000017) | <.001 | | | | |  | | | |  | | | | 0.0001396(0.000021) | | | | <.001 | | | |  | | | |  | | |
|  | **Family income ≤ 150000 CNY (n=65)** | | | | | | | | | | | | | | | **Family income ＞ 150000 CNY (n=41)** | | | | | | | | | | | | | |
|  | **Coefficient (SE)** | | | | ***p* value** | | | **SD (SE)** | | | | ***p* value** | | | | **Coefficient (SE)** | | | | ***p* value** | | | | **SD (SE)** | | | | ***p* value** | |
| Second-tier city | 0.660 (0.178) | | | | <.001 | | | 0.737(0.272) | | | | .007 | | | | 1.267(0.374) | | | | .001 | | | | 0.923(0.474) | | | | <.001 | |
| First-tier city | 1.234 (0.238) | | | | <.001 | | | 1.227(0.267) | | | | <.001 | | | | 2.380(0.521) | | | | <.001 | | | | 1.966(0.486) | | | | <.001 | |
| Housing allowance provided | 0.492 (0.161) | | | | .002 | | | 0.246(0.364) | | | | .50 | | | | 0.387(0.274) | | | | .16 | | | | 0.278(0.476) | | | | .56 | |
| Housing provided | 0.816 (0.165) | | | | <.001 | | | 0.080(0.641) | | | | .90 | | | | 0.623(0.263) | | | | .02 | | | | 0.106(0.560) | | | | .85 | |
| Children’s education opportunities: Good | 0.468 (0.112) | | | | <.001 | | | 0.090(0.431) | | | | .83 | | | | 0.970(0.301) | | | | .001 | | | | 1.241(0.372) | | | | .001 | |
| Career promotion speed: 3 year | 0.141(0.152) | | | | .35 | | | 0.158(0.395) | | | | .69 | | | | 0.537(0.281) | | | | .06 | | | | 0.482(0.392) | | | | .22 | |
| Career promotion speed: 1 year | 0.531 (0.152) | | | | <.001 | | | 0.309(0.379) | | | | .42 | | | | 0.795(0.326) | | | | .02 | | | | 1.058(0.438) | | | | .02 | |
| Working environment: Better | 0.155 (0.114) | | | | .17 | | | 0.340(0.203) | | | | .09 | | | | 0.360(0.193) | | | | .06 | | | | 0.196(0.356) | | | | .58 | |
| *bianzhi:* Offer | 0.869 (0.156) | | | | <.001 | | | 0.679(0.175) | | | | <.001 | | | | 0.841(0.286) | | | | .003 | | | | 1.184(0.371) | | | | .001 | |
| Income | 0.0000964(0.000014) | | | | <.001 | | |  | | | |  | | | | 0.0001574(0.000029) | | | | <.001 | | | |  | | | |  | |

CNY: Chinese yuan; Abbreviation: SD, standard deviation; SE: standard error; : US$1 = CNY 6.901 (2020).
